# Recognizing employees’ contribution to effectiveness and values: A randomized waitlist-controlled trial of operant-based leadership training

**DOI:** 10.1371/journal.pone.0320131

**Published:** 2025-04-24

**Authors:** Martin Grill

**Affiliations:** Department of Psychology, University of Gothenburg, Gothenburg, Sweden; Sichuan Agricultural University, CHINA

## Abstract

To develop and sustain a healthy and efficient work environment, managers need to provide their employees with relevant and useful performance feedback. However, research on leadership training in functional behaviors, such as performance feedback, has yet to demonstrate consistent long-term effectiveness. Therefore, the purpose of this study was to evaluate the long-term influence of operant-based leadership training on managers’ performance feedback behaviors. Municipality employees (*n* = 439) responded to a questionnaire four times over a period of 18 months. The employees’ managers were randomized into an experimental and a control group. The managers in the experimental group received managerial behavioral training, while the waitlist control group managers did not. Multilevel growth curve modeling was used for data analysis. The results showed that employees in the experimental group reported a significantly greater improvement in their managers’ performance feedback behaviors compared to employees in the control group (*β* = 0.30, *p* = 0.04). Furthermore, the improvement in the experimental group managers’ leadership behaviors had a linear trajectory throughout the 18-month study period, with no significant deceleration in their learning curve (Δ*χ*^2^(2) = 2.2, *p* = 0.34). In conclusion, operant-based leadership training can help managers develop their performance feedback behaviors and the effect of such training is persistent over time. The findings imply that leadership training research and practice should strive to integrate operant learning theory and practice in order to improve the long-term effectiveness of leadership training interventions. Positive utility reactions, achieved by shaping leadership behaviors to optimize their fit with the managers’ work context, may be critical for ensuring the enduring effects of leadership training.

## Introduction

Functional managerial leadership is an important part of healthy and efficient work environments. Managers’ leadership behaviors have been found to have medium to large (*d* = 0.46–0.98) long-term influence on employees’ motivation and productivity [1]. One fundamental component of functional managerial leadership is providing employees with appropriate and relevant performance feedback [2]. Effective performance feedback is in high demand [3] and involves acknowledging employees’ contributions to fulfilling organizational goals (i.e., effectiveness-related performance feedback) [4], as well as contributions to other important organizational and societal values (i.e., values-related performance feedback) [5]. However, the relevance and quality of the performance feedback managers give their employees vary considerably [6], which can substantially alter the impact of performance feedback [4]. Effective feedback involves being specific about how the employees’ contributions help the organization achieve specific organizational and/or societal goals, it provides employees with recognition and improves their role clarity [7]. Hence, to uphold healthy and efficient work environments, managers need training in how to provide employees with relevant and useful performance feedback related to both effectiveness and values.

Meta-analyses indicate that interventions for leadership development tend to yield favorable outcomes in the short term but that more research is needed on methods that promote lasting improvements in managers’ leadership behaviors [8,9]. In addition, the findings are inconclusive because most leadership training research lacks randomized controlled trials and rarely includes long-term follow-up measures [10]. Furthermore, intensive longitudinal research indicates that the initial positive effects of leadership training may begin to diminish as early as six weeks after the start of the training [11]. In summary, leadership training research through randomized controlled trials on how to accomplish lasting improvements in managers’ leadership behaviors has been called for [10,12–16]. Hence, the aim of this study was to evaluate the long-term influence of operant-based leadership training on managers’ effectiveness-related and values-related performance feedback behaviors.

### Performance feedback and operant-based leadership training

Performance feedback allows employees to develop their skills, improve their work performance, and produce better quality outputs [17]. However, despite the availability of numerous studies, reviews, and meta-analyses, a consensus has not been reached on the processes or outcomes associated with feedback, nor on how performance feedback skills are most efficiently trained [17–20]. These challenges have contributed to the conclusion that feedback is a complex and multifaceted topic [17,21], prompting calls for further investigation into what feedback entails and how it is most effectively performed and trained [4].

Performance feedback has been a long-standing area of focus within organizational behavior, management, and leadership research [17]. The roots of performance feedback research can be traced back to the operant learning theory of Skinner [22], who concluded that “The importance of feedback is clear, the organism must be stimulated by the consequences of its behavior if conditioning is to take place.” (p. 67). According to operant learning theory, positive reinforcement is a fundamental mechanism for the influence of performance feedback on employee behavior [17].

Managerial leadership can be defined as managing the reinforcement contingencies that influence the behavior of employees [23], and influencing others is central to most definitions of leadership [24]. Yukl and colleagues [25,26] researched managers’ leadership strategies for influencing employees and found rational persuasion and inspirational appeal to be two of the most successful. That is, employee performance tends to improve when managers use logical arguments, factual evidence, and appeals to employees’ values and ideals, to communicate the feasibility and relevance of their work in achieving important task objectives. For performance feedback, this implies using logical arguments and factual evidence when acknowledging employees’ contributions to achieving organizational goals (i.e., effectiveness-related performance feedback) [4] and explicitly communicating to employees how they contribute to important organizational and societal values (i.e., values-related performance feedback) [5].

This study aspires to advance the field of performance feedback and leadership training research by evaluating if managers’ performance feedback behaviors can be developed through an operant-based leadership training intervention. While existing research has provided valuable insights into the effectiveness of performance feedback for employee performance, the next step is to understand how managers’ performance feedback behaviors can be developed.

The difficulty in achieving enduring effects from leadership training has led to increased interest in factors that may help or hinder managers in implementing and maintaining the leadership behaviors developed during training interventions [27]. Operant learning theory provides a framework for understanding when and how trained behaviors are sustained or discarded by conceptualizing training as bringing performers into contact with existing natural sources of instrumental and social reinforcement [28,29]. However, research into how operant learning theory can be applied to generate lasting improvement in managers’ leadership behaviors is still lacking. Leadership behaviors are shaped through continuous interactions between managers and their employees in which positive reinforcement occurs for behaviors that are followed by beneficial consequences [30]. Hence, sustained effect of leadership training is more likely if the leadership behaviors trained are mutually rewarding for the managers and their employees. Based on operant learning theory, Grill et al. [31] found that managers’ positive utility reactions in the workplace were associated with their learning curves during leadership training. Similarly, Tafvelin et al. [32] reported that managers’ positive utility reactions to the trained behaviors in their work environment were an important success factor in leadership training. However, to what extent positive utility reactions continue to positively reinforce managers’ leadership behaviors after the training has terminated remains undetermined.

Operant-based leadership training involves applying procedures to optimize operant learning experiences: that is, shaping contextually adaptive leadership behaviors (i.e., behaviors that fit the managers’ work environment and employees) and providing managers with ample opportunities to train—and monitor the positive consequences of—the targeted behaviors [31,33,34]. Interventions grounded in operant learning theory are typically based on needs analyses [35] and comprise behavior analyses, goal setting, and practice with behavioral feedback [28,29,33]. Behavior analysis is an operant learning operationalization of behavior that takes into account significant and controllable contextual stimuli that are functionally linked to behavioral deficits and/or accesses [36]. Performed in collaboration with a manager and trainer, it can be used to identify and understand behavioral deficits and/or accesses in the manager’s current leadership practice [31,34] and to determine how these deficits and/or accesses are functionally related to the behaviors of the manager’s employees—that is, how the manager’s behaviors function as reinforcing contingencies for employee behaviors [37]. The behavioral deficits and/or accesses identified in the behavior analyses can be used to formulate individualized behavioral goals [36,38] and targeted through practice with behavioral feedback. Practice with behavioral feedback typically involves in-session behavior rehearsal [39] while the trainer provides behavior-specific feedback on performance, as well as between-session behavioral training in everyday interactions at work through homework assignments (i.e., implementing behaviors and enhancing performance across different work situations where the behavior proves useful) [40]. Accordingly, the operant-based leadership training tested in the present study included behaviors analysis, goal setting, and practice with behavioral feedback.

Improvements often continue after the end of interventions based on operant learning theory; that is, individuals tend to further develop and generalize functional behaviors on their own [35,41]. Managers in operant-based leadership training have reported positive utility reactions in the form of positive responses from employees (i.e., social reinforcers) as well as stress reduction, time-use efficiency, and successful organizational change (i.e., instrumental reinforcers) [31,34,42]. Hence, behaviors developed through operant learning leadership training are likely to be positively reinforced as managers continue to perform them in their everyday work environment after training has ended. Such positive utility reactions should lead to sustained learning and continuous use of the trained leadership behaviors.

*Hypothesis:* Managers’ effectiveness-related and values-related performance-feedback behaviors can be enduringly improved through operant-based leadership training.

## Methods

### Participants and procedure

This study was approved by the Swedish Ethical Review Authority (reg. no. 1060-18/2019–00590). Written consent was obtained from all participants. Managers in local government organizations were recruited from a municipality in Sweden between March 1 and June 1, 2019; 106 managers and their employees were invited to participate in the study, and 59 managers and 583 of their employees agreed to participate. The types of local government organizations in the study included education, healthcare, construction and development, property management and maintenance, water and waste management, social and emergency services, elderly care, transportation, administration, economics, cultural administration, tourism, communication, and human resource management. These organizations encompass the typical work contexts of Swedish municipal employees and managers. The managers were randomized into an experimental group (*n* = 30) who received leadership training and a waitlist control group (*n* = 29) who received the leadership training after all data had been collected. The randomization was performed by the principal investigator of the research project without having any previous knowledge about the participants. The RAND function in Microsoft Excel (v. 16) was used to assign each manager with a random decimal number between 0 and 1. The managers were subsequently ranked according to this random number and the first 30 managers were placed in the experimental group while the last 29 were allocated to the waitlist control group. The 30 managers in the experimental group were then randomly assigned to trainers for leadership training, with each of the five trainers responsible for six managers. Only the managers and trainers were notified of the randomization results. To ensure blinding of those evaluating the dependent variables (i.e., the managers’ employees), the managers were instructed not to disclose any information about the training to their employees, colleagues, or superiors. Employees were informed that the study aimed to evaluate leadership training and that 50% of the managers would undergo the training. They were also told about the blinding procedure: “To avoid influencing your responses with any expectations of leadership changes, you will not be informed whether or not your manager is receiving training.”

An online questionnaire was distributed to the managers’ employees at four timepoints at 6-month intervals: pre-measure in June–July 2019 (T1); post-measure in December 2019–January 2020 (T2); 6-month follow-up in June–July 2020 (T3); and 12-month follow-up in December 2020–January 2021 (T4). In total, 500 employees (74%) responded to the questionnaire on one or more occasions (69% at T1, 69% at T2, 68% at T3, and 62% at T4). During the study, five managers quit their job or discontinued their training due to lack of time; another five managers focused their training on behaviors others than performance feedback and were hence excluded from the analysis. The final sample consisted of *n* = 178 employees from *n* = 20 managers in the experimental group and *n* = 261 employees from *n* = 29 managers in the control group. The characteristics of the participants are shown in Table 1.

**Table 1:** Characteristics of the participants.

Characteristic	Experimental group			Control group			Total		
	M	SD	%	M	SD	%	M	SD	%
Manager characteristics									
Age (in years)	47.0	5.3		45.0	5.2		45.8	5.3	
Years of managerial experience	8.0	6.3		7.2	6.8		7.5	6.5	
University graduate			100			89			94
Gender (female)			60			59			60
Management level^a^									
Operational manager			75			74			74
Executive manager			25			26			26
Employees’ characteristics									
Age (in years)	45.5	10.7		44.8	10.8		45.1	10.7	
Tenure with manager (in years)	1.6	1.9		1.7	3.8		1.7	3.2	
Gender (female)			63			64			63
University graduate			80			76			78
Organization type									
Administration			28			29			29
Education			28			13			20
Health services			12			25			18
Arts and culture			8			4			6
Miscellaneous			24			29			27

*Note*. M, mean; SD, standard deviation. The experimental group consisted of *n* = 178 employees and *n* = 20 managers; the control group consisted of *n* = 261 employees and *n* = 29 managers. ^a^Operational managers do not have managers reporting to them, whereas executive managers do.

### Managerial behavioral training

The managerial behavioral training (MBT) manual [43] was used to train the managers in the experimental group over a period of 3 months, from September to November 2019. The MBT comprised six one-on-one, face-to-face, biweekly 90-minute sessions consisting of behavior analysis, goal setting, and practice with performance feedback.

The trainer and manager jointly conducted the behavior analysis, which consisted of the identification and analysis of antecedent and consequent stimuli functionally related to their leadership behaviors (i.e., analyzing the topography and function of the behavior), including their performance feedback behaviors. Throughout the behavior analysis process, the managers received training in analyzing both their own behaviors and those of their employees [44]. Via the behavior analysis, the manager and trainer collaboratively identified the manager’s most significant behavioral deficiencies. Specific goals were then established to address these deficiencies. One to three behavioral [45] and participative (i.e., agreed upon by the manager and trainer) [46] goals were defined and documented (e.g., “I will hold weekly follow-up meetings with all employees to give them feedback on their performance”). Training exercises for different leadership behaviors are detailed in the 11 elective modules of the MBT manual from which the manager and the trainer jointly selected the one to three modules that best suited the manager’s behavioral goals. The modules covered validation, handling criticism, managing employee behaviors, positive reinforcement through feedback, delivering corrective feedback, time management, managing meetings, setting and following up on goals, problem-solving, cognitive reappraisal, and mindfulness [43].

The main training activity consisted of practice with performance feedback through in-session behavioral rehearsal [39]. In this way, the managers’ leadership behaviors were shaped in accordance with their needs and goals. The managers were recorded during the exercises and provided with video feedback [47] on their performance. Homework assignments [48] were formulated and documented at the conclusion of each session. These tasks involved implementing the leadership behavior practiced during the session within the manager’s work setting to ensure that it fitted with the manager’s work environment. At the start of the subsequent training session, the homework was reviewed, and the manager received performance feedback on behaviors aligned with their behavioral goals.

### Measures

The managers’ performance feedback behaviors were measured using the eight items from the performance feedback and value-based performance feedback scales developed by Grill et al. [34], based on the work of Yukl et al. [26]. The four effectiveness-related performance feedback items are based on the four-item rational persuasion scale, and the four value-based performance feedback items are based on the four-item inspirational appeal scale [26]. The wording of the items and their factor loadings from a one-factor confirmatory factor analysis were as follows: “My manager…” “…describes how my work is contributing to organizational objectives” (*β* = 0.85–0.90 at T1–T4), “…clarifies how my work is contributing to organizational performance and effectiveness” (*β* = 0.88–0.93), “…provides information or evidence that demonstrates the significance of my work” (*β* = 0.83–0.88), “…uses facts and logic when describing the significance of my work” (*β* = 0.85–0.90), “…describes my work as meaningful and important” (*β* = 0.86–0.88), “…describes valuable objectives obtained through my work” (*β* = 0.85–0.90), “…describes how my work contributes to important values and ideals” (*β* = 0.87–0.92), and “…talks about the importance of my work” (*β* = 0.85–0.90). All items were rated on a Likert scale ranging from 1 (*Never*) to 7 (*Always*).

Factorial invariance was assessed using the Lavaan package [49] in R (v. 2023.03.1), following Cheung and Rensvold’s [50] change in comparative fit index (ΔCFI) < 0.01 criteria for comparing models with no constraints (configural invariance), models with constrained item factor loadings (weak invariance), and models with both constrained item factor loadings and item intercepts (strong invariance). For time invariance, the ΔCFI was <.01 in all stepwise comparisons of the configural invariant model (CFI =.931), the weak invariant model (CFI =.931), and the strong invariant model (CFI =.930). For group invariance across the experimental and control groups, the ΔCFI was <.01 in all stepwise comparisons of the configural invariant model (CFI =.916), the weak invariant model (CFI =.916), and the strong invariant model (CFI =.916). Hence, the performance feedback measure demonstrated strong factorial invariance across both time and groups.

### Data analysis

For hypothesis testing, multilevel growth curve modeling (MLM) [51] with the lme4 package in R (v. 2023.03.1) was used in accordance with the work of Finch et al. [52]. The models comprised all three levels in the data: repeated observations (level 1) from employees (level 2) nested within managers (level 3). The variation at each level was accounted for by random effects of intercept and time. Random effects allow for the intercept and slope to vary across clusters and thereby accommodate the non-independence between repeated observations nested within the same employee (level 2) and the multiple employees nested within the same manager (level 3). Time was coded from 0 to 1 (i.e., 0 for T1, 0.333 for T2, 0.666 for T3, and 1 for T4). Maximum likelihood (ML) was used for the estimations and for handling missingness in data [53]; thus, listwise deletion could be avoided and data from all respondents were appropriately weighted according to their contribution at each time point. The total number of observations were 1126, corresponding to 35.9% missing data. The proportion of missing data was 25.7% at T1, 32.8% at T2, 39.6% at T3, and 45.3% at T4.

The effect of the training was tested by comparing a model with main effects of training and time (Model 1) with a model that also included the interaction effect between training and time (Model 2). To test whether the effect of training was stable across time, a third model that also included a curvilinear main effect of time and an interaction effect between the curvilinear effect of time and training (Model 3) was estimated and compared with Model 2. The models were compared using Δχ^2^ tests. The dependent variable was standardized (*m* = 0, *sd* = 1) prior to data analysis so that the estimated parameters correspond to effects sizes equivalent to Cohen’s *d* [54].

## Results

**Fig 1:**
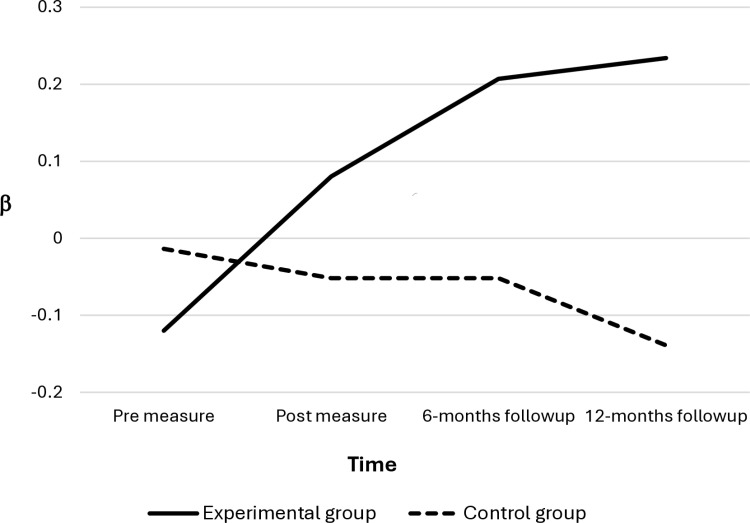
The standardized means of the managers’ performance feedback behaviors at T1 to T4.

Table 2 provides descriptive statistics and intercorrelations for the performance feedback variable at each time point for the experimental and the control group. The results of the MLMs are provided in Table 3, and the trajectories over time in the mangers’ performance feedback behaviors are depicted in Fig 1. Model 2—including the interaction effect of time and training—was found to have a significantly better model fit (Δ*χ*^2^ (1) = 4.4, *p* = 0.04) compared with the baseline model (Model 1). This result implies that the change in performance feedback over time was conditioned on the training; that is, employees with managers in the experimental group reported a more positive time-bound change in their managers’ performance feedback behaviors than employees in the control group (*β* = 0.30, *p* = 0.04). Model 3, which included a curvilinear effect of time and its interaction with the effect of the training, was not found to have a significantly better model fit (Δ*χ*^2^ (2) = 2.2, *p* = 0.34) than Model 2. This result implies that the change in performance feedback did not deviate from a continuous linear trajectory; that is, the increase in managers’ performance feedback due to the training did not decline over time. The results support the hypothesis that managers’ effectiveness-related and values-related performance feedback behaviors were enduringly improved through the operant-based leadership training.

**Table 2:** Descriptive statistics and intercorrelations for performance feedback at T1–T4.

		Experimental group				Control group							
		M	SD	α	ICC	M	SD	α	ICC	1.	2.	3.	4.
1.	Performance feedback T1	4.64	1.32	0.96	0.20	4.75	1.48	0.96	0.26		0.80	0.68	0.66
2.	Performance feedback T2	4.91	1.30	0.96	0.16	4.73	1.33	0.96	0.24	0.71		0.73	0.75
3.	Performance feedback T3	5.09	1.35	0.97	0.15	4.73	1.40	0.96	0.13	0.71	0.75		0.74
4.	Performance feedback T4	5.12	1.25	0.96	0.19	4.61	1.55	0.98	0.21	0.55	0.73	0.82	

*Note*. Means (M), standard deviations (SD), Cronbach’s alphas (α), and intraclass correlations (ICC) for the experimental (*n* = 178) and control group (*n* = 261) employees. Intercorrelations are shown for the experimental group below the diagonal and for the control group above the diagonal.

**Table 3:** Results from the MLM describing the change in performance feedback from T1 to T4.

	Model 1				Model 2				Model 3			
	*β*	*SE*	*p*	95% CI	*β*	*SE*	*p*	95% CI	*β*	*SE*	*p*	95% CI
Fixed effects												
Intercept	-0.044	0.102	0.664	-0.252–0.162	0.003	0.104	0.978	-0.203–0.212	0.006	0.105	0.953	-0.202–0.217
Time	0.123	0.070	0.088	-0.019–0.266	-0.010	0.093	0.911	-0.206–0.176	-0.048	0.195	0.805	-0.433–0.335
Training	0.050	0.154	0.745	-0.277–0.374	-0.059	0.163	0.718	-0.386–0.265	-0.089	0.164	0.590	-0.418–0.237
Time^*^training					0.297	0.140	0.040^*^	0.020–0.600	0.622	0.296	0.036^*^	0.041–1.207
Quadratic time									0.041	0.180	0.820	-0.313–0.395
Quadratic time ^*^training									-0.347	0.276	0.209	-0.891–0.196
Random effects												
Level 2 Intercept	0.747			0.673–0.828	0.747			0.673–0.828	0.748			0.673–0.828
Level 2 Time	0.548			0.385–0.699	0.553			0.391–0.704	0.553			0.391–0.704
Level 3 Intercept	0.457			0.333–0.613	0.457			0.335–0.612	0.455			0.333–0.609
Level 3 Time	0.275			0.063–0.456	0.268			0.059–0.443	0.263			0.047–0.437
Residuals	0.469			0.439–0.501	0.467			0.438–0.500	0.467			0.438–0.500
Model fit												
Deviance	2531.1				2526.7				2524.5			
Df	10				11				13			
Δ*χ*^2^					4.4^a^				2.2^b^			
Sig-test of Δ*χ*^2^					0.036^*^				0.336			

Note. The experimental group consisted of *n* = 178 employees and the control group of *n* = 261 employees. Model 1 estimated the effects of time and training on performance feedback. Model 2 estimated the interaction effect between time and training (i.e., conditioning the time-bound change in performance feedback on the training). Model 3 estimated a curvilinear effect of time and its interaction with the effect of training.

^a^Compared to model 1

^b^Compared to model 2.

* *p* < 0.05.

## Discussion

The purpose of this research was to address employees’ need for relevant and high-quality performance feedback related to both effectiveness and values, as part of a healthy and efficient work environment. By acknowledging employees’ contributions to fulfilling organizational goals and other important organizational and societal values, managers can provide their employees with recognition and improve role clarity. To this end, the effectiveness of operant-based leadership training for enduring improvements in managers’ performance feedback behaviors was evaluated. The findings indicate that the effect of the training on managers’ effectiveness-related and values-related performance feedback behaviors was persistent for the 18-month duration of the study. Randomized controlled field trials in leadership training research are rare, and finding sustained positive effects of such training instills some confidence that leadership training may be a worthwhile investment in employees’ psychosocial work environment. Employees’ need for relevant and high-quality performance feedback may partly be met by providing managers with operant-based leadership training interventions.

The learning mechanism employed in MBT involved exposing managers to individualized instrumental and social reinforcers through the application of behavior analysis, goal setting, and practice with performance feedback. Adapting leadership training to managers’ contextual circumstances is not unique for operant-based leadership training; a meta-analysis by Lacerenza et al. [9] showed that pre-assessing managers’ individual needs and providing managers with feedback were general success factors for leadership training. However, in MBT, individualization and performance feedback are not only used in the initial part of the training but are continuously integrated throughout the training.

The results of this study indicate that the quality of managers’ feedback can be improved by combining feedback training with training in behavior analysis. When managers use behavior analysis to identify the consequences of employees’ behaviors and how employees contribute to organizational and societal goals–and specify how this information can be communicated to their employees–the feedback is more likely to be relevant and useful for employees. Teaching behavior analysis to managers enables them to provide their employees with behavior-specific performance feedback tailored to individual behaviors, needs and goals. Developing managers’ behavior-analytic skills can also enhance their ability to provide reinforcers for desirable employee behaviors beyond positive feedback, further improving employee performance through a range of social and instrumental reinforcers. These results align with the findings from operant learning safety-leadership training research by Gravina et al. [33] and Grill et al. [31]. The practical applicability of the findings includes helping managers understand how employee behavior is influenced by its consequences and how they can make sure positive consequences are provided for positive employee behaviors. Applying behavior analysis in leadership practice is an implementable recommendation for professionals involeved in leadership and leadership training.

Operant learning theory stipulates that the learning of social behaviors is dependent on their social validity and that enduring behaviors are shaped through mutually rewarding interactions between individuals [55,56]. Managers’ effectiveness-related and values-related performance feedback behaviors can produce positive consequences for both managers and employees. When managers provide employees with performance feedback, employees’ goal-oriented performance tends to increase [18]—a desired consequence for most managers and, therefore, a likely positive reinforcer for them. When employees receive performance feedback, they experience recognition and improved role clarity [3,7], which are likely positive reinforcers for them. Managers’ functional leadership behaviors can be formed in mutual interactions between managers and employees in such a way that leadership behaviors with positive utility for both parties are more likely to be enduring in the long-term. Recent longitudinal research corroborates that managers’ positive leadership behaviors are, in part, shaped by their employees [57,58].

The other two central components of the training in this study were goal setting and practice with behavioral feedback. Previous research on goal setting shows that the effect of goal setting is amplified when goals are firmly grounded in a thorough needs analysis and jointly formulated by the trainer and the trainee [46]. Ludwig and Geller [46] examined the impact of assigned versus participative goal setting, concluding that participative goals are more effective for response generalization. Specifically, when individuals participated in setting their own behavioral goals, they improved not only the targeted behavior but also related, non-targeted behaviors. Leadership behaviors are inherently complex, involving multiple stimulus–response chains, requiring advanced problem-solving and decision-making, and influencing employee performance and work output both directly and indirectly [5,37,59,60]. This complexity suggests that response generalization—and by extension, participative goal setting—may play a crucial role in leadership training.

Practice with behavioral feedback is an important component of efficient training, well-established in both training theory and practice [28]. However, in leadership training, practice with behavioral feedback is often underutilized. Most leadership training research focus predominately on targeting cognitive processes through lectures, discussions, and reflections [8,9]. Indeed, it can be challenging to isolate specific behaviors in leadership practice that are suitable for behavioral rehearsal [60]. However, the findings in this study suggest that incorporating extensive practice with behavioral feedback into leadership training programs is a practicl and implementable recommendation for professionals involved in leadership and leadership development, in order to achieve lasting effects. In the precent study, a considerable proportion of the practice with behavioral feedback took place at the managers’ workplaces as they completed their homework assignments. Previous research on other types of operant learning-based interventions has found that homework assignments constitute a significant proportion of their effect (d=0.48) [40]. On-site training activities has also been identified as an important positive moderator in Lacarenzas [9] meta-analysis of leadership training.

### Limitations

The leadership training was participative; that is, it was based on collaborative analyses of each participant’s pre-training leadership behaviors, needs, and goals. Hence, the intervention was tailored to train each manager in behaviors specifically relevant for them. An inherent challenge for participative interventions is ensuring rigor in evaluation [61]. Training tailored to individual needs inevitably results in different managers being trained in different behaviors; therefore, not all managers randomized to the experimental group were trained in performance feedback behaviors. However, for leadership training to be effective, it must align with the specific needs of the individual manager and most managers did address performance feedback in their training. Insufficient sample size precluded analyses of other trained behaviors; a larger sample size was planned for, but the COVID-19 pandemic interrupted a second batch of managers halfway through their training. In future participative intervention research, larger sample sizes are needed to evaluate each participant on measures optimally suited for their specific individualized goals. Such designs will provide more accurate effect size estimates and improve precision in comparisons between participative interventions and one-size-fits-all interventions.

COVID-19 developed into a pandemic between the post-measurement (T2) and the 6-month follow-up (T3), significantly impacting all sectors of society, including the organizations in this study. Lockdowns imposed during this period restricted physical interaction between employees and their managers, necessitating a shift toward online communication methods for collaboration and leadership. In addition, managers found themselves increasingly consumed by troubleshooting, as crisis meetings became a routine response to emerging challenges. This situation prompted a notable reevaluation of priorities, with leadership development taking a back seat to more pressing concerns. Consequently, the extent to which the findings of this study can be applied to post-COVID societies remains uncertain. It is likely that the influence of leadership training on managers’ leadership behaviors is more pronounced during periods with fewer crisis-related disruptions. Furthermore, as this study was conducted within the context of Swedish municipalities, the extent to which the findings generalize to other societal sectors and national cultures should be addressed in future research.

### Conclusion

Operant-based leadership training through MBT can help managers develop relevant effectiveness-related and values-related performance feedback behaviors, and the effect of the training is sustained over time. Leadership training should be individualized and include behaviors analysis, goal setting, and practice with behavioral feedback. Favorable utility reactions and mutually rewarding manager-employee interactions may play an important role in sustaining the long-term effectiveness of leadership training interventions. The findings of this study suggest that researchers, professionals, and policymakers involved in leadership training research and practice should incorporate principles and methods from operant learning theory to provide a more comprehensive understanding and enhance the long-term effectiveness of leadership development programs.
